# Association between MRI-based tumor-to-skin distance and axillary lymph node metastasis in breast cancer

**DOI:** 10.55730/1300-0144.6359

**Published:** 2026-05-11

**Authors:** Hikmet PEHLEVAN ÖZEL, Seçil GÜNDOĞDU, Eda ŞAHİNGÖZ, Ebru MENEKŞE, Mesut TEZ

**Affiliations:** 1 Department of General Surgery, Ankara Bilkent City Hospital, Ankara, Turkiye; 2 Department of Radiology, Ankara Bilkent City Hospital, Ankara, Turkiye

**Keywords:** Breast cancer, tumor-to-skin distance, axillary lymph node metastasis, magnetic resonance imaging, prognostic marker

## Abstract

**Background/aim:**

Axillary lymph node (ALN) status is a major prognostic factor in breast cancer and influences both survival outcomes and treatment planning. Noninvasive predictors of nodal metastasis are of clinical interest. Tumor-to-skin distance (TSD) has been proposed as a potential anatomical marker; however, standardized data based on magnetic resonance imaging (MRI) remain limited.

**Materials and methods:**

This retrospective study included 98 female patients with pathologically confirmed breast cancer who underwent preoperative breast MRI between 2020 and 2025. Patients with prior breast or axillary surgery, distant metastasis, multifocal or multicentric disease, or inadequate MRI quality were excluded. TSD was measured on dynamic contrast-enhanced MRI by a single experienced radiologist blinded to axillary nodal status. Associations between TSD and histopathologically confirmed ALN metastasis were assessed using logistic regression analysis. Diagnostic performance was evaluated by receiver operating characteristic (ROC) analysis.

**Results:**

Among the 98 patients, 27 (27.6%) had pathologically confirmed ALN metastasis. The median TSD was significantly shorter in the ALN-positive group than in the ALN-negative group (10.0 mm versus 14.0 mm, p = 0.004). In multivariate analysis, TSD was independently associated with nodal metastasis (OR, 0.929; 95% CI 0.864–0.999; p = 0.048). ROC analysis demonstrated moderate discriminatory ability (AUC = 0.69). A cutoff value of 10 mm yielded a sensitivity of 62.9%, specificity of 73.2%, and a high negative predictive value of 83.9%.

**Conclusion:**

A shorter TSD measured on preoperative breast MRI is independently associated with ALN metastasis. A 10-mm threshold may help identify patients at low risk of nodal involvement, supporting MRI-based TSD measurement as a complementary tool for preoperative risk stratification. Prospective multicenter studies are warranted for validation.

## 1. Introduction

Breast cancer is the most common malignancy in women and one of the leading causes of cancer-related death worldwide [1]. Axillary lymph node (ALN) status is one of the most important prognostic factors, directly influencing both survival outcomes and surgical and adjuvant treatment strategies [2]. Therefore, identifying reliable preoperative predictors of nodal involvement is crucial for optimizing surgical planning, avoiding overtreatment, and individualizing adjuvant therapy.

Several studies have suggested that, in addition to tumor size and histological features, anatomical parameters such as tumor-to-skin distance (TSD) and tumor-to-nipple distance may be associated with axillary metastasis. Ansari et al. demonstrated that tumor proximity to the skin and nipple is associated with a higher likelihood of ALN metastasis [3]. Yang et al. reported that the tumor-to-nipple distance is independently associated with ALN metastasis [4]. Yur et al. reported that ALN metastasis is significantly more common in patients with a shorter tumor-to-skin distance [5]. Collectively, these findings suggest that tumor proximity to superficial anatomical structures may reflect an anatomical correlate of axillary nodal involvement rather than a direct biological mechanism of metastatic spread.

Despite these observations, most previous studies have relied on ultrasonography or intraoperative and pathological measurements, which are prone to operator dependence and limited reproducibility. Magnetic resonance imaging (MRI), with its high spatial resolution and multiplanar capabilities, allows a more accurate and reproducible assessment of tumor-to-skin distance. However, data regarding MRI-based TSD and its association with ALN involvement remain limited.

Accordingly, the present study aimed to investigate the association between MRI-measured tumor-to-skin distance and axillary lymph node metastasis in patients with breast cancer and to identify a statistically derived cutoff value that may assist in preoperative risk stratification. We hypothesized that a shorter tumor-to-skin distance measured on preoperative breast MRI would be independently associated with the presence of axillary lymph node metastasis.

## 2. Materials and methods

### 2.1. Study design

This single-center retrospective cohort study was conducted at a tertiary referral hospital between January 2020 and January 2025. The study was approved by the institutional ethics committee (approval no: 2-25-1028/2025) and was conducted in accordance with the Declaration of Helsinki.

A total of 527 patients with pathologically confirmed breast cancer were initially screened. The inclusion criteria were female sex, age ≥18 years, histologically confirmed invasive breast carcinoma, and availability of preoperative breast MRI. The exclusion criteria were prior breast or axillary surgery, distant metastasis at diagnosis, a previous history of breast cancer, multifocal or multicentric tumors, absence of a measurable mass on MRI, and significant image artifacts precluding reliable measurement.

Only patients who underwent preoperative breast MRI and were subsequently referred directly to surgery without neoadjuvant therapy were included. All patients underwent sentinel lymph node biopsy (SLNB) as the initial axillary staging procedure. Completion axillary lymph node dissection (ALND) was performed selectively in patients with positive SLNB findings or clinically/radiologically suspicious axillary involvement, in accordance with current guideline-based deescalation strategies (e.g., ACOSOG Z0011 criteria) [2]. Axillary lymph node (ALN) status was defined on the basis of histopathological evaluation of sentinel lymph node biopsy and/or axillary lymph node dissection specimens. Patients with histopathologically confirmed axillary lymph node metastasis detected at surgery were classified as node-positive. Patients referred to neoadjuvant therapy without histopathological confirmation of nodal status were excluded.

**Figure 1:**
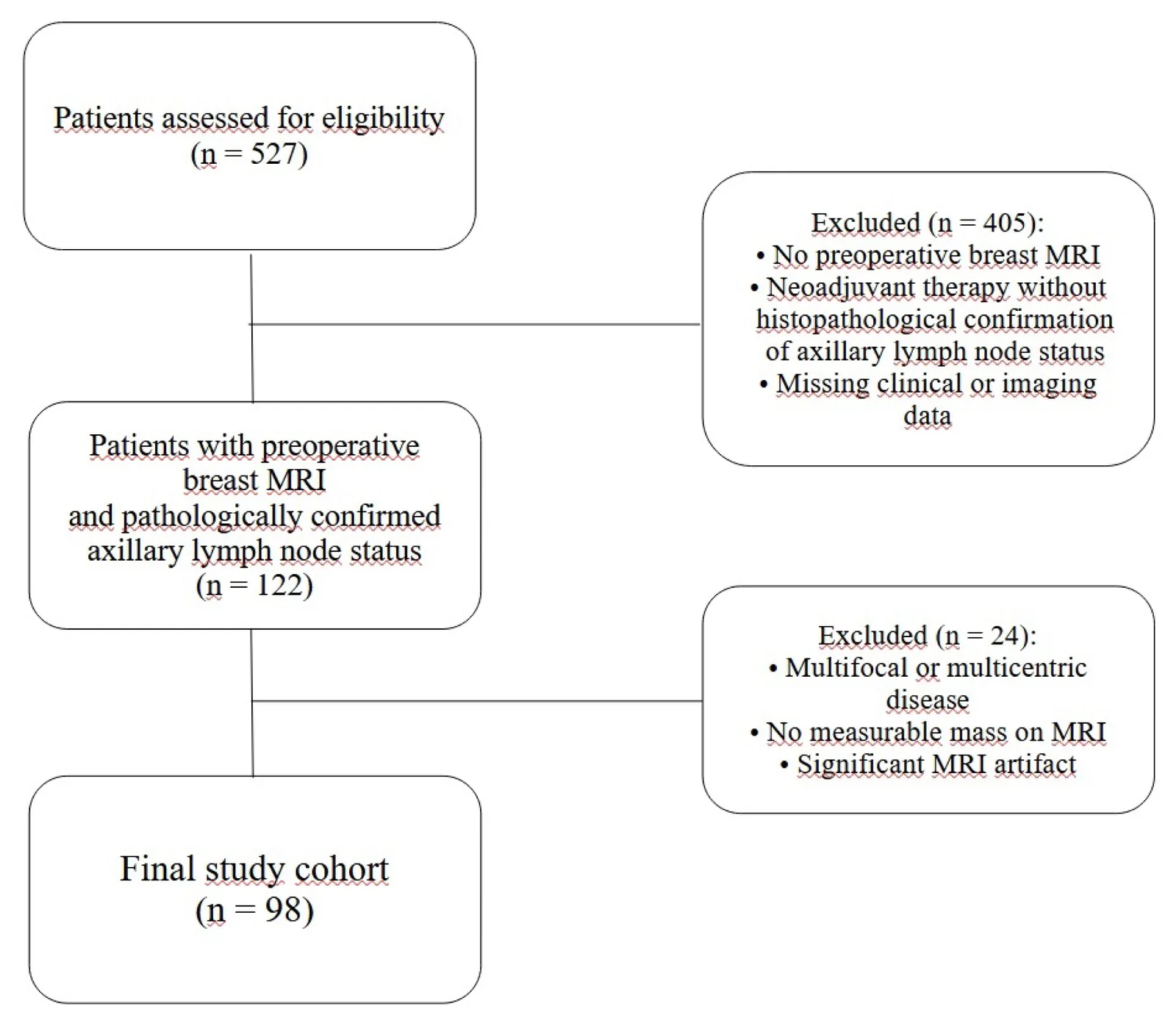
Flowchart of patient selection.

After applying these criteria, 98 patients were included in the final analysis (Figure 1). No formal sample size calculation was performed because of the retrospective design of the study, and all consecutive eligible patients during the study period were included.

### 2.2. Data collection

Demographic, clinical, radiological, and pathological data were retrieved from institutional electronic medical records. The collected variables included age, menopausal status, family history, tumor location, calcifications, hormone receptor status, the Ki-67 proliferation index, histological tumor grade (according to the Nottingham grading system), lymphovascular invasion, perineural invasion, accompanying ductal carcinoma in situ, MRI tumor size, tumor-to-nipple distance, and tumor-to-skin distance.

### 2.3. MRI acquisition and measurements

All breast MRI examinations were performed using a 3-T MRI system (Signa Pioneer, General Electric Medical Systems, Milwaukee, WI, USA), with patients positioned prone in a 16-channel phased-array bilateral breast coil. Dynamic contrast-enhanced axial T1-weighted fat-suppressed sequences were obtained following intravenous administration of a gadolinium-based contrast agent (0.2 mmol/kg), which was injected through a 22-gauge intravenous catheter and followed by a 20 mL saline flush. Tumor-to-skin distance and tumor-to-nipple distance were measured on high-resolution postcontrast images and corresponding subtracted images using electronic calipers. Measurements were defined as the shortest distance (in millimeters) between the tumor margin and either the external dermal boundary (skin surface) or the nipple base, as assessed on multiplanar reconstruction images, most commonly in the oblique sagittal or oblique axial plane. All measurements were performed by a single radiologist with 10 years of experience in breast imaging, blinded to axillary lymph node status. To minimize measurement bias, each measurement was obtained at the point of the shortest distance between the tumor margin and the skin surface.

### 2.4. Statistical analysis

Statistical analyses were performed using SPSS software (version 21.0; IBM Corp., Armonk, NY, USA). Continuous variables were expressed as mean ± standard deviation or median (minimum–maximum), depending on data distribution, while categorical variables were presented as frequencies and percentages. Group comparisons were conducted using the independent samples t-test or Mann–Whitney U-test for continuous variables and the chi-square test for categorical variables. Normality was assessed using the Kolmogorov–Smirnov test. Logistic regression analysis was performed to identify factors associated with axillary lymph node involvement. Variables with a p < 0.20 in the univariate analysis were included in the multivariate logistic regression model using the backward likelihood ratio method. Prior to multivariable modeling, collinearity among candidate variables was assessed, and no variable exhibited variance inflation factors exceeding commonly accepted thresholds. The diagnostic performance of tumor-to-skin distance was evaluated using receiver operating characteristic (ROC) curve analysis. The optimal cutoff value was determined using Youden’s index. The area under the curve (AUC), sensitivity, specificity, positive predictive value, and negative predictive value were calculated. A p < 0.05 was considered statistically significant, and all analyses were conducted using a 95% confidence interval (CI).

## 3. Results

A total of 527 patients with breast cancer were screened during the study period. After the inclusion and exclusion criteria had been applied, 98 patients were included in the final analysis (Figure 1).

All patients underwent sentinel lymph node biopsy (SLNB) as the initial axillary staging procedure. Axillary surgery consisted of SLNB alone in 92 patients (93.9%), SLNB followed by completion axillary lymph node dissection (ALND) in four patients (4.1%), and upfront ALND in two patients (2.0%). Completion ALND was not routinely performed in all SLNB-positive patients, in accordance with contemporary deescalation strategies.

Among these, 27 patients (27.6%) had pathologically confirmed ALN metastasis, while 71 (72.4%) were node-negative. The mean age was 56.1 ± 10.3 years in the ALN-positive group and 54.1 ± 10.6 years in the ALN-negative group; the difference was not statistically significant (p = 0.394). Most clinicopathological and imaging variables, including menopausal status, family history, tumor size, tumor-to-nipple distance, Ki-67 index, human epidermal growth factor receptor 2 status, and histological grade, were not significantly associated with nodal status. Detailed clinicopathological and imaging characteristics stratified by axillary lymph node involvement are presented in Table 1. On MRI, the median tumor-to-skin distance (TSD) was significantly shorter in patients with ALN metastasis than in node-negative patients (10.0 mm [0.1–25.0] versus 14.0 mm [1.0–85.0], p = 0.004).

In univariate logistic regression, estrogen receptor (ER) percentage (OR, 1.020; 95% CI 1.002–1.038; p = 0.030), progesterone receptor (PR) positivity (OR, 8.185; 95% CI 1.033–64.888; p = 0.047), and TSD (OR, 0.922; 95% CI 0.861–0.987; p = 0.020) were significantly associated with ALN metastasis. In multivariable analysis, TSD remained independently associated with ALN metastasis (OR, 0.929; 95% CI 0.864–0.999; p = 0.048), whereas PR positivity (p = 0.060) and the presence of ductal carcinoma in situ (p = 0.075) showed borderline statistical significance (Table 2). This finding indicates that each 1 mm increase in tumor-to-skin distance was associated with an approximately 7% reduction in the odds of axillary lymph node metastasis.

**Figure 2:**
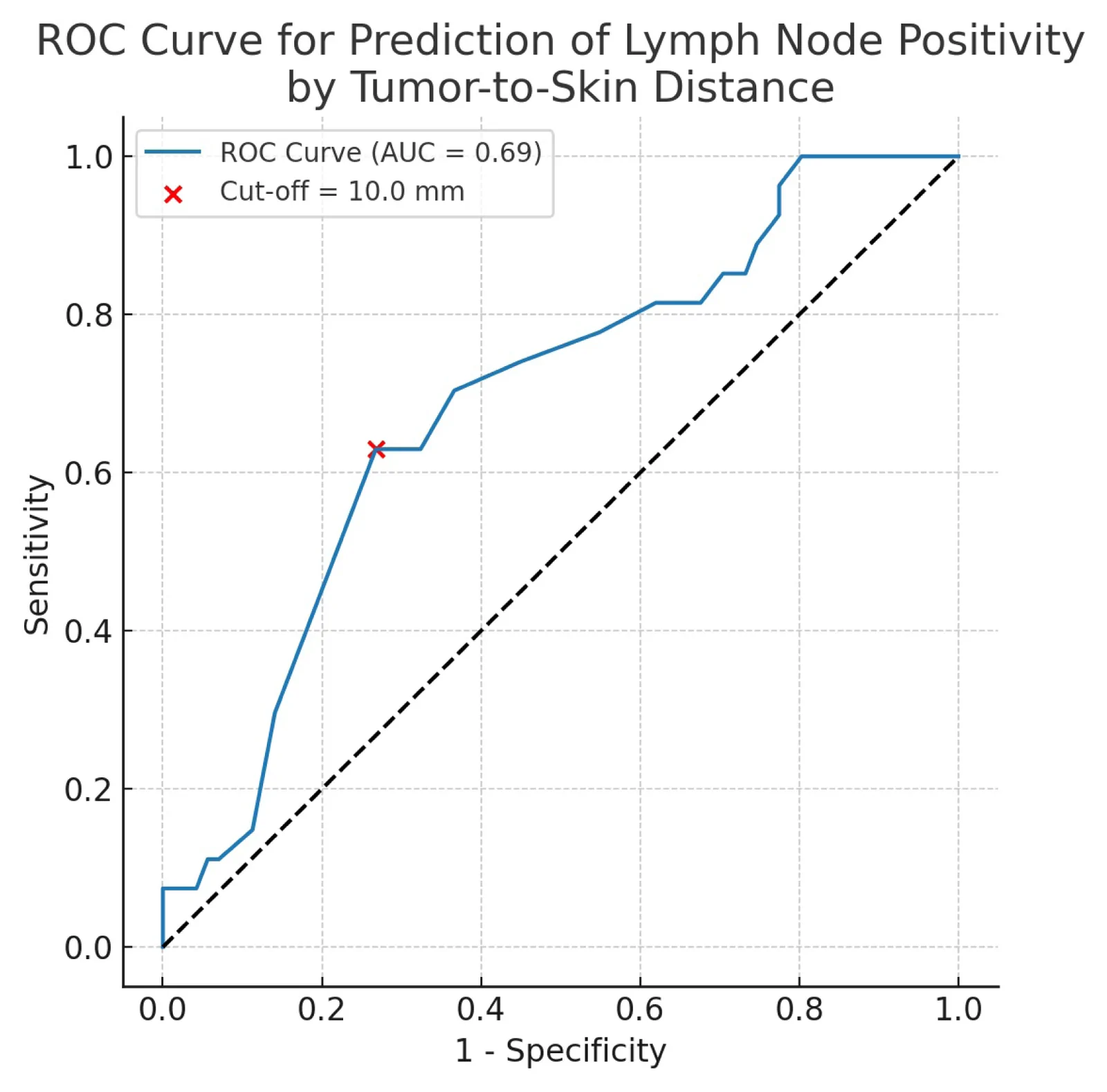
ROC curve of tumor-to-skin distance for prediction of axillary lymph node metastasis. Receiver operating characteristic (ROC) curve illustrating the diagnostic performance of tumor-to-skin distance for predicting axillary lymph node metastasis in patients with breast cancer. The analysis yielded an area under the curve (AUC) of 0.69, indicating moderate discriminatory ability. Using a cutoff value of 10 mm determined by Youden’s index, tumor-to-skin distance demonstrated a sensitivity of 62.9%, a specificity of 73.2%, an accuracy of 70.4%, a positive predictive value of 47.2%, and a negative predictive value of 83.9%, using histopathologically confirmed nodal status as the reference standard.

ROC curve analysis demonstrated that TSD had moderate discriminatory ability for predicting ALN metastasis, with an AUC of 0.69 (Figure 2). The ROC analysis was performed using histopathologically confirmed nodal status derived from SLNB and/or ALND as the reference standard. Using a cutoff value of 10 mm determined by Youden’s index, the sensitivity was 62.9%, the specificity was 73.2%, the accuracy was 70.4%, the positive predictive value was 47.2%, and the negative predictive value was 83.9% (Table 3). Notably, the high negative predictive value suggests that patients with a tumor-to-skin distance greater than 10 mm are unlikely to have axillary nodal metastasis.

## 4. Discussion

This study evaluated the association between tumor-to-skin distance (TSD) and axillary lymph node (ALN) metastasis in patients with breast cancer. The findings demonstrated that a shorter TSD was significantly associated with nodal positivity and that MRI-based TSD remained independently associated with ALN involvement after adjustment for other clinicopathological factors. The 10-mm cutoff value derived from ROC analysis provided moderate discriminatory performance (AUC = 0.69) and a high negative predictive value (83.9%), highlighting the potential utility of TSD primarily as a rule-out parameter rather than a standalone predictor of nodal disease.

Axillary lymph node involvement is a well-established determinant of prognosis in breast cancer and plays a central role in guiding both surgical and adjuvant treatment strategies. Large population-based studies have consistently demonstrated that nodal positivity is associated with significantly reduced survival, independent of primary tumor size [6]. Similarly, the Early Breast Cancer Trialists’ Collaborative Group (EBCTCG) reported increased mortality and a greater benefit from systemic therapy among patients with lymph node metastases [7]. Recent randomized studies have also emphasized that axillary nodal status directly impacts survival and treatment decisions [2]. In this context, identifying imaging-based parameters that may refine preoperative nodal risk assessment remains clinically relevant.

The results of the present study are consistent with previous reports indicating an association between tumor proximity to superficial anatomical structures and axillary metastasis. Ansari et al. found that tumors closer to the skin and nipple were more likely to metastasize to axillary nodes [3]. Yang et al. confirmed that tumor-to-nipple distance is an independent predictor of nodal positivity [4]. Similarly, Yur et al. demonstrated that shorter TSD was significantly associated with ALN metastasis [5]. These findings should be interpreted as an anatomical and imaging-based correlate of nodal involvement rather than evidence of a direct causal mechanism.

In addition to anatomical factors, biological and histopathological characteristics are known to influence nodal involvement. In the present study, ER percentage and PR positivity were associated with nodal metastasis in univariate analysis; however, these associations did not retain statistical significance after multivariable adjustment. Kim et al. reported that triple-negative breast cancers show distinct localization patterns, linking tumor subtype and nodal involvement [8]. The EBCTCG metaanalysis further demonstrated that nodal positivity has more substantial prognostic implications in hormone receptor–negative tumors [7]. At the molecular level, processes such as lymphangiogenesis, epithelial–mesenchymal transition, chemokine signaling, and immune evasion have been shown to promote lymphatic spread, reinforcing the mechanistic plausibility of these findings [9]. Within this framework, TSD may represent an imaging-derived parameter that complements, rather than replaces, established biological predictors of nodal metastasis.

Preoperative imaging remains central to nodal risk assessment in breast cancer. Ultrasonography and mammography are widely used but are limited by operator dependence and spatial resolution. Breast MRI provides high-resolution multiplanar evaluation and allows reproducible measurement of tumor location relative to surrounding anatomical structures. Kuijs et al. reported in their systematic review that MRI alone may not reliably exclude nodal metastasis but can be valuable in combination with other modalities [10]. Marino et al. emphasized MRI’s role in superficial tumors and morphologic evaluation [11]. Cai et al. demonstrated that MRI combined with ultrasound yields moderate diagnostic accuracy but cannot replace sentinel lymph node biopsy [12]. Consistent with these findings, the moderate AUC observed in the present study supports the role of TSD as an adjunctive rather than definitive diagnostic parameter.

Importantly, the clinical relevance of these findings should be interpreted in the context of contemporary axillary management. In our cohort, all patients underwent SLNB as the initial staging procedure, and completion ALND was performed selectively rather than routinely in all node-positive patients, reflecting current guideline-based deescalation strategies [2]. This evolving paradigm underscores the need for reliable preoperative markers that may assist in identifying patients with a low likelihood of nodal disease, thereby potentially reducing unnecessary axillary interventions.

The strengths of this study include the use of standardized preoperative 3-T dynamic contrast-enhanced MRI, blinded assessment by an experienced breast radiologist, and histopathological confirmation of nodal status. However, several limitations should be acknowledged. The retrospective single-center design introduces potential selection bias, and the relatively small sample size may limit statistical power. Interobserver variability of MRI-based measurements was not assessed, which may affect reproducibility across institutions. In addition, the study population consisted exclusively of patients who underwent primary surgery without neoadjuvant therapy, which may limit the generalizability of the findings to broader breast cancer populations. Furthermore, given that ALND was not uniformly performed in all node-positive patients, a degree of nodal understaging cannot be entirely excluded. Finally, the moderate discriminatory performance observed suggests that tumor-to-skin distance should be interpreted as a complementary imaging parameter rather than a definitive predictor of ALN metastasis.

This study demonstrated that tumor-to-skin distance measured on preoperative breast MRI was independently associated with axillary lymph node metastasis in patients with breast cancer. A 10-mm threshold derived from ROC analysis showed moderate discriminatory ability with a high negative predictive value, indicating that tumors located farther from the skin are less likely to be associated with nodal involvement. Accordingly, MRI-based assessment of tumor-to-skin distance may serve as a complementary imaging parameter to support preoperative risk stratification, rather than as a standalone criterion for axillary management.

Further multicenter prospective studies with larger cohorts are warranted to validate these findings and to clarify the potential role of tumor-to-skin distance within multimodal predictive frameworks for axillary evaluation.

## Figures and Tables

**Table 1:** Distribution of demographic, clinical, magnetic resonance imaging, and pathological characteristics according to lymph node involvement status.

Characteristics	Lymph node involvement		p-value

	Absence of lymph node (n = 71, 72.4%)	Presence of lymph node (n = 27, 27.6%)	

Age, years, mean ± SD	54.06 ± 10.59	56.07 ± 10.28	0.394

Menopausal status, n (%)			
Absence	19 (67.9%)	9 (32.1%)	0.520
Presence	52 (74.3%)	18 ( 25.7%)	

Family history, n (%)			
Absence	53 (72.5%)	20 (27.4%)	0.940
Presence	18 (72.0%)	7 (28.0%)	

Location, n (%)			
Periareolar	3 (42.9%)	4 (57.1%)	
Upper Right Quadrant	21 (67.7%)	10 (32.3%)	
Lower Right Quadrant	14 (100%)	0	
Upper Left Quadrant	27 (67.5%)	13 (32.5%)	0.011^*^
Lower Left Quadrant	6 (100%)	0	

**Microcalcification**, n (%)			
Absence	36 (75%)	12 (25%)	0.580
Presence	35 (70%)	15 (30%)	

ER status, n(%)			
Negative	15 (93.8%)	1 (6.3%)	0.062
Positive	56 (68.3%)	26 (32.5%)	

ER, %, median (minimum-maximum)	90 (0–100)	95 (0–100)	0.016

PR status, n(%)			
Negative	17 (94.4%)	1 (5.6%)	0.020^*^
Positive	56 (67.5%)	26 (32.5%)	

PR, %, median (minimum-maximum)	57.50 (0–100)	80 (0–100)	0.085

HER2 status, n(%)			
Negative	58 (73.4%)	21 (26.6%)	0.662
Positive	13 (68.4%)	6 (31.6%)	

Ki-67, median (minimum-maximum)	20 (0–95)	20 (3–90)	0.813

Nottingham grade score, median (minimum-maximum)	6.0 (0–9.0)	6.0 (4.0–9.0)	0.986

Grade, n (%)			
Grade 1	13 (65.0%)	7 (35.0%)	
Grade 2	42 (72.4%)	16 (27.6%)	0.767
Grade 3	12 (75.0%)	4 (25%)	

Lymphovascular invasion, n (%)			
Absence	46 (79.3%)	12 (20.7%)	0.067
Presence	25 (62.5%)	15 (37.5%)	

Perineural invasion, n (%)			
Absence	59 (76.6%)	18 (23.4%)	0.077
Presence	12 (57.1%)	9 (42.9%)	

DCIS status, n (%)			
Absence	17 (89.5%)	2 (10.5%)	0.064
Presence	54 (68.4%)	25 (31.6%)	

MRI tumor size, mm, median (minimum-maximum)	20.0 (7.0–61.0)	23.0 (12.0–44.0)	0.325

Tumor-to-nipple distance, mm, mean ± SD	58.56 ± 29.84	54.04 ± 31.12	0.519

Tumor to skin distance, mm, median (minimum-maximum)	14.0 (1.0–85.0)	10.0 (0.1–25.0)	0.004

SD: standard deviation; ER: estrogen receptor; PR: progesterone receptor; HER2: human epidermal growth factor receptor 2; Ki-67: Ki-67 proliferation index; DCIS: ductal carcinoma in situ; MRI: magnetic resonance imaging.

**Table 2:** Factors associated with lymph node involvement: results of univariate and multivariable logistic regression analyses.

	Univariate Analysis		Multivariate Analysis	
	OR (95% CI)	p-value	OR (95% CI)	p-value
ER expression, %	1.020 (1.002–1.038)	0.030		
ER positivity	6.964 (0.893–55.578)	0.067		
PR expression, %	1.012 (0.999–1.024)	0.061		
PR positivity	8.185 (1.033–64.888)	0.047		0.060
Presence of lymphovascular invasion	2.300 (0.933–5.668)	0.070		
Presence of perineural invasion	2.458 (0.893–6.768)	0.082		
Presence of DCIS	3.935 (0.844–18.355)	0.081		0.075
Tumor-to-skin distance	0.922 (0.861–0.987)	0.020	0.929 (0.864–0.999)	0.048

ER: estrogen receptor; PR: progesterone receptor; DCIS: ductal carcinoma in situ; OR: odds ratio; CI: confidence interval.

Variables with p < 0.20 in the univariate analysis were included in the multivariable logistic regression model using the backward likelihood ratio method.

**Table 3:** Diagnostic performance of tumor-to-skin distance (cut-off: 10 mm) for predicting lymph node positivity.

Tumor-to-skin distance	Lymph node positive	Lymph node negative	Total
≤10 mm	17	19	36
>10 mm	10	52	62
Total	27	71	98
**Performance metrics** Accuracy: 70.4% Sensitivity: 62.9% Specificity: 73.2% Positive predictive value: 47.2% Negative predictive value: 83.9%			

## Data Availability

The data used and/or analyzed during the current study are available from the corresponding author upon reasonable request.
